# Binder materials for the cathodes applied to self-stratifying membraneless microbial fuel cell

**DOI:** 10.1016/j.bioelechem.2018.04.011

**Published:** 2018-10

**Authors:** Xavier Alexis Walter, John Greenman, Ioannis Ieropoulos

**Affiliations:** Bristol BioEnergy Centre, Bristol Robotics Laboratory, T-Block, Frenchay Campus, University of the West of England (UWE), Bristol BS16 1QY, United Kingdom

## Abstract

The recently developed self-stratifying membraneless microbial fuel cell (SSM-MFC) has been shown as a promising concept for urine treatment. The first prototypes employed cathodes made of activated carbon (AC) and polytetrafluoroethylene (PTFE) mixture. Here, we explored the possibility to substitute PTFE with either polyvinyl-alcohol (PVA) or PlastiDip (CPD; i.e. synthetic rubber) as binder for AC-based cathode in SSM-MFC. Sintered activated carbon (SAC) was also tested due to its ease of manufacturing and the fact that no stainless steel collector is needed. Results indicate that the SSM-MFC having PTFE cathodes were the most powerful measuring 1617 μW (11 W·m^−3^ or 101 mW·m^−2^). SSM-MFC with PVA and CPD as binders were producing on average the same level of power (1226 ± 90 μW), which was 24% less than the SSM-MFC having PTFE-based cathodes. When balancing the power by the cost and environmental impact, results clearly show that PVA was the best alternative. Power wise, the SAC cathodes were shown being the less performing (≈1070 μW). Nonetheless, the lower power of SAC was balanced by its inexpensiveness. Overall results indicate that (i) PTFE is yet the best binder to employ, and (ii) SAC and PVA-based cathodes are promising alternatives that would benefit from further improvements.

## Highlights

• Activated carbon based cathodes were investigated in SSM-MFCs.
• PTFE showed to have the higher power production.
• Binder concentration did not affect directly the power production.
• PVA-based cathodes and sintered activated carbon were the most cost effective.

## 1 Introduction

Microbial Fuel Cells (MFCs) is a technology able to degrade organics from waste streams and produce electricity simultaneously. The utilisation of biocatalysts (i.e. microorganisms) for the direct production of electricity from organics was demonstrated firstly in 1911 [1]. A MFC is generally composed of an anode and a cathode in which oxidation and reduction reactions occur. Anodophillic microorganisms on the anode degrade organics and generate an electron flow through the external load, whilst oxygen reduction reaction (ORR) occurs at the cathode, completing the red-ox reactions. Over the recent years, research focused on improving the reactor configuration and size [2,3], identifying electrodes [4,5] and separator materials [6], and assembling collectives of units into stacks [7] (i.e. electrical and hydraulic connections). These improvements led the technology to the point of being implemented at pilot scale: microbial electrolysis cell for hydrogen production from wastewater [[8], [9], [10]], benthic microbial fuel cells [11,12], MFC in constructed wetland for wastewater treatment [[13], [14], [15]], prototype to be integrated in wastewater treatment plant [[16], [17], [18], [19], [20], [21], [22]], and MFC-based urinal system [23].

As a single MFC is intrinsically a low power source, a plurality of MFC must be assembled in stacks to produce exploitable power levels [24,25]. In parallel, applied MFC research focuses on cost-effective materials as well as simplicity of design whilst maximising power production (i.e. high power density of a single MFC). Recently, the concept of self-stratifying membraneless MFC (SSM-MFC) has been shown to address such a dual need [26]: SSM-MFC have a high power densities and a simple design removing the need for complex/expensive construction processes. It was shown that an SSM-MFC can be scaled-up in size, up to a certain extent, without significant power density losses [26]. SSM-MFC relies on the ability of microorganisms to vertically self-stratify the physicochemical conditions of any given water column. In undisturbed natural environments (e.g. lakes), chemical gradients will develop under the activity of biological populations. As a result, the environment gets divided in horizontal layers, each characterised by specific bio-chemical conditions (i.e. redox state of chemical elements, type of dominating metabolic activity). In lakes for example, this stratification phenomenon result in the upper layers of the water column being aerobic and the lower layers being anaerobic [27]. This natural phenomenon is exploited for electricity production by placing the cathodes in the upper layer of such columns (i.e. aerobic environment), whilst placing the anodes in the bottom layer (i.e. anaerobic and reduced environment). In this configuration, both electrodes a submerged in the same electrolyte and part of the cathode exposed directly to air [26]. The physicochemical gradient separating the upper layer of the urine column from the bottom layer could, thus, be considered a transient membrane reformed after each feeding pulses disturbance. Details on SSM-MFCs principle and properties are described in an earlier study conducted by Walter et al. [26].

Urine is a very complex biofluid that may contain up to 2651–3079 different metabolites [28] each with a specific biodegradation path. However, they can be encompassed in the broad category of urea, organic acids, creatinine, amino acids and carbohydrates. We assume that urea accounts for about 50% of the organic carbon contained in urine and that it is usually subject to partial hydrolysis by the naturally occurring microflora. Urea ((NH_2_)_2_CO) is primarily hydrolysed by urease enzyme into ammonia (NH_3_) and carbamate (NH_2_COOH), which is subsequently degraded to ammonia and carbon dioxide [29]. When treated through microbial fuel cell systems, most of these organic compounds get degraded in the anodic compartment [30]. Hence, we can assume such organic degradation is also occurring in the anode part of SSM-MFCs which can be represented by the acetate oxidation Eq. (1), as an exemplary of such reactions:

(1) $${\mathrm{CH}}_{3}{\mathrm{COO}}^{-}+4H_{2}O\to 2{\mathrm{HCO}}_{3}^{-}+9H^{+}+8e^{-}$$

The cathodic reactions involved in the functioning of SSM-MFCs are yet to be determined. The cathodic reactions could involve the reduction of oxygen and/or oxidised nitrogen, in the form of, for example, nitrate or nitrite. However, urine typically contains low amounts of nitrate and nitrite and they would be the product of the autotrophic ammonium oxidation processes that involve oxygen, as represented by Eqs. (2a), (2b):

(2a) $${\mathrm{NH}}_{4}^{+}+1.5O_{2}\to {\mathrm{NO}}_{2}^{-}+H_{2}O+2H^{+}$$

(2b) $${\mathrm{NO}}_{2}^{-}+0.5O_{2}\to {\mathrm{NO}}_{3}^{-}$$

Under these conditions, the autotrophic microorganisms involved in the aerobic oxidation of ammonium would be competing for oxygen. This agrees with most microbial fuel cell systems employing activated carbon based cathodes, in which it is the oxygen 2e^−^ reduction mechanism that occurs, as represented by Eq. (3) for neutral and alkaline conditions [31,32]:

(3) $$O_{2}+H_{2}O+2e^{-}\to {\mathrm{HO}}_{2}^{-}+{\mathrm{OH}}^{-}$$

Studies have demonstrated that NO_3_^−^ could be reduced into N_2_ on an anaerobic cathode (denitrification), whilst a simultaneous nitrification was occurring on a separated aerobic cathode [[33], [34], [35]]. In the case of SSM-MFCs, we speculate that both reactions are taking place along the cathode, with the nitrification occurring in the higher (aerobic) section whilst the denitrification would occur in the lower (anaerobic) section of the cathodes. If these phenomena were occurring in SSM-MFC, they would contribute towards the demonstration that SSM-MFCs are cost-effective systems for nitrogen recycling. However, the processes involved in the cathodic reactions of SSM-MFC are yet to be determined. Hence, characterising the exact redox reactions occurring in SSM-MFC is an aspect that would need further detailed investigations.

Nonetheless, the concept of SSM-MFC is as a cost-effective system worth developing further. Up to now, this research concept was developed by our group [26] as a power source for remote area [36,37]. To further develop this concept, the present study focused on the cathode development with the aim of: i) decreasing the production costs; ii) finding alternative materials to substitute components of the electrodes that are not environmentally friendly (e.g. polytetrafluoroethylene (PTFE)). Therefore, the investigation focused on a range of cathode binder materials that were either inexpensive and/or environmentally friendly and/or had simplified production processes. Amongst the inexpensive and high performing cathode materials, activated carbon(AC)/PTFE mixture applied on a mesh acting as the current collector (i.e. nickel [38] or stainless steel [26]) is one of the main used cathode for MFCs [26,39]. In this work, polyvinyl alcohol (PVA) was also employed as a potential binder because of its biocompatibility and the advantage of (i) being rigid once dried, and (ii) employing water as solvent prior being fixed at 180 °C. In parallel, also PlastiDip was investigated as a binder because (i) it is rapid to produced (dry time ≈ 1 h), and (ii) the finish product is rigid (i.e. robustness of the system). A control based on AC and PTFE as binder was also used. After fabrication, the cathodes were operated in SSM-MFC fed with human urine and polarisation and power curves were presented and discussed. At last, another material that was of interest due to its ease of manufacturing and low cost was sintered activated carbon (SAC), which is mainly used for water filtration and is essentially found as cylindrical monolithic cartridges.

## 2 Materials and method

### 2.1 Tested cathodes material

All the tested materials were using AC as catalyst (Table 1). The main differences investigated were due to the utilization of diverse binders (Table 1). Because of the nature of sintered activated carbon (SAC), the cathodes were directly fixed on the supporting bolts. The SAC cathodes were cut (≈2–3 mm thick) from water filter cartridges (Water Filterman, UK). The SAC dimensions were 45 mm × 100 mm (10.62 ± 0.44 g). The manufacturer of the SAC water filtration cartridge employed high molecular weight polyethylene (HMW-PE) as a binder. No specification in terms of HMW-PE type and weight percentage was provided. The AC/PTFE cathodes had a stainless-steel mesh backbone (316SS, 8 × 8 mesh; 8.32 ± 0.15 g; MeshDirect, UK) acting as the current collector on which was applied the AC/PTFE mixture (final weight of 16.70 ± 0.46 g; AC/PTFE loading of 186 ± 7 mg·cm^−2^) (Table 1). This catalytic mixture was prepared as previously described [26,36,40]. Both the polyvinyl alcohol (PVA; Sigma-Aldrich, UK) and PlastiDip (CPD; PlastiDip, UK) based cathodes also had a stainless-steel mesh backbone. The PVA-10 (i.e. PVA 10%wt) mix comprised 90 g activated carbon powder and 200 mL water-based 5% PVA (w/v) stock solution (final weight of 10.92 ± 0.43 g; AC/PVA loading of 58 ± 7 mg.cm^−2^). The PVA-5 (i.e. PVA 5%wt) mix comprised 47.2 g activated carbon powder and 50 mL water-based 5% PVA (w/v) stock solution final (weight of 12.43 ± 0.65 g; AC/PVA loading of 91 ± 11 mg.cm^−2^). Once applied onto the stainless-steel mesh, the paste was dried overnight. Once dried, the PVA cathodes were placed in an oven at 180 °C for 1 h. The paste applied on the stainless-steel mesh for the CPD-5 (i.e. CPD 5%) cathodes consisted of 19 g activated carbon powder mixed to 2.5 g of PlastiDip and 20 mL of solvent (petroleum ether 80°–110°) – 2.5 g of fresh PlastiDip has a dry weight of ≈1 g. The CPD paste was rapidly applied and then put 1 h in the fume hood for the solvent to evaporate (both the PlastiDip solvent and the added petroleum ether). The CPD cathodes final weight was of 19.48 ± 0.77 g, which correspond to an AC/CPD loading of 248 ± 14 mg cm^−2^. Although the loadings between the cathodes are rather different, the objective here was not to compare the mixture from a quantitative perspective (i.e. at similar loading), but from an implementation perspective. In fact, the cathodes needed to have those specific dimensions (45 mm large, 100 mm in length and 3 mm thick) to fit a specific design adopted during the experimentation. Hence, the difference in loadings is the result of the production procedures.

**Table 1:** Main characteristics of the material tested as cathodes in SSM-MFCs.

Denomination	Support/current collector	Catalyst	Loading (mg·cm^−2^)
PTFE	Stainless steel mesh	AC powder	186 ± 7
SAC	N/A	Sintered AC powder	N/A
PVA-10	Stainless steel mesh	AC powder	58 ± 7
PVA-5	Stainless steel mesh	AC powder	91 ± 11
CPD-5	Stainless steel mesh	AC powder	248 ± 14

The cathodes materials (Table 1) were tested through 2 successive experimental batches, all with the same SSM-MFC design (see Section 2.2) and each time new cathodes and new anodes: 1st batch experiments: PTFE, PVA-10; 2nd batch experiments: PTFE, PVA-5, CPD-5.

### 2.2 SSM-MFC construction and operations

**Fig. 1:**
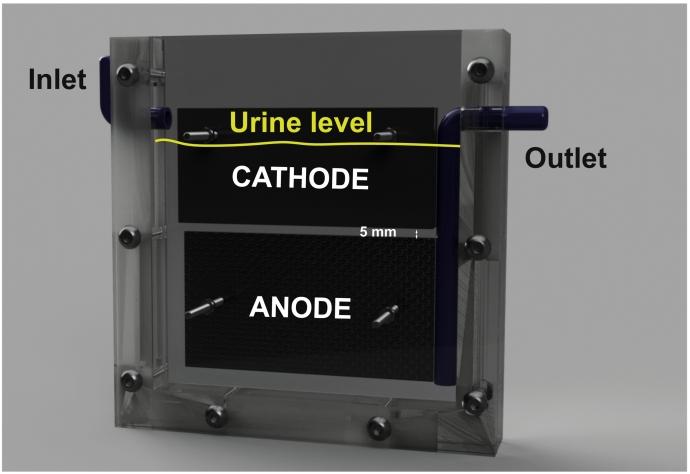
3D representation of the SSM-MFC design. Both cathodes and anodes are submerged in the same electrolyte. Part of the cathode has to be open to air [26].

The MFC embodiment consisted of a 15 mm thick U-shaped core sandwiched between two 5 mm thick acrylic plates (Fig. 1). Two U-shaped 1.5 mm thick silicon gaskets were maintaining water-tightness of the MFC. Two flat cathodes (45 mm in height, 100 mm in length and 3 mm thick) were suspended 5 mm above two flat anodes. These anodes were fabricated using 624 cm^2^ carbon fibre veil (20 g·m^2^, PRF Composite Materials Poole, Dorset, UK) that was folded down to a projected surface area of 50 cm^2^ (50 mm × 100 mm). Stainless steel 316 M3 bolts were maintaining the electrodes in position and maintaining a good physical contact with them, whilst protruding 20 mm from the side. These bolts, which serve as current collectors, were also used to connect the monitoring cables and the resistive loads using crocodile clips. All the electrodes were submerged in the same electrolyte, which in the present study was undiluted human urine. In every case, 10 mm of the cathode heights were emerging above the urine level (Fig. 1) [26]. 50 mL of fresh urine was pulse-fed every 3 h at the top of the liquid column whilst the same amount was retrieved from the bottom of the liquid column. The average displacement volume was 143 ± 7 mL and the hydraulic retention time was ≈9 h for every MFC.

Human urine (pH between 6.4 and 7.4) was collected and pooled daily, from anonymous and healthy individuals with no known previous medical conditions. As the urine was kept in a tank (max. 24 h), it had already gone through partial hydrolysis by naturally occurring microflora. Hence, the pH and solution conductivity of the urine feeding the MFCs had values varying between 8.3 and 8.9 and 24–28 mS·cm^−1^ respectively. Human urine broadly comprises 4.7–10.4 g·L^−1^ dry matter of which 65–85% are organic compounds, with urea being the main constituent of these total organic solids (≈50%) [41]. The SSM-MFCs were inoculated with a mixture comprising 50% (v/v) of the output stream of a matured MFC (running under identical conditions and also fuelled with urine), and 50% (v/v) of freshly collected urine. The SSM-MFC were then run under a constant 500 Ω load for 3–4 days, then 300 Ω and finally 100 Ω load.

### 2.3 Data capture and polarisation experiments

Voltage outputs were monitored over time using an Agilent LXI 34972A data acquisition/switch unit (Farnell, UK). Data were recorded every 3 min. The polarisation scan was performed on mature modules (i.e. modules had reach electrical steady-state). The polarisation sweeps were performed using an automated resistorstat [42] with resistive load values ranging from 10,000 Ω to 21 Ω. Each resistive load (12) was applied for a period of 15 min. The current *–* in Amperes (A) – was calculated according to the Ohm's law, *I* = *V*/*R*, where *V* is the measured voltage in Volts (V) and *R* is the known value of the resistor. The power output *P* in Watts (W) was calculated as *P* = *I* × *V.*

## 3 Results and discussion

Due to its high porosity, AC is not amongst the most conductive carbon materials. Usually carbon black (CB) is added to the mixture to increase the electrical conductivity of the cathode material and therefore the overall performance [43,44]. However, a long-term experiment (5 months) has shown that the performance of AC cathodes were only 17% lower than cathode incorporating 10%wt CB [44]. Because of that and due to its petrochemical origin, no CB was added to any of the tested mixtures.

**Fig. 2:**
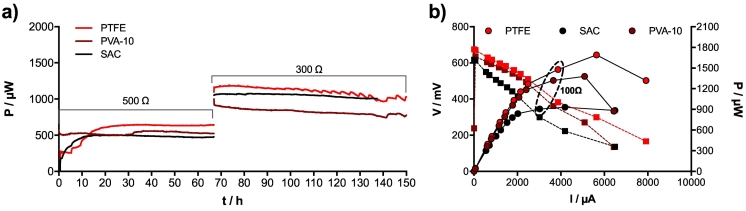
Electrical performance of the SSM-MFC mounted with the tested cathodes. a) Temporal evolution of the power produced under 2 different loads. b) Results from the polarisation experiment carried at *t* = 150 h (a).

Results show that under 500 Ω and 300 Ω, the sintered activated carbon cathodes (SAC) were producing similar level of power as the PTFE (≈1150 μW) and slightly more than the PVA-10 cathodes (≈850 μW; Fig. 2a). Conversely, results from the polarisation experiment indicate that PTFE and PVA-10 cathode could potentially produce, more power (Fig. 2b). These results imply that the SSM-MFC mounted with SAC were already under an optimum load (i.e. 300 Ω) corresponding to their maximum power transfer point whereas the SSM-MFC mounted with PTFE and PVA-10 cathodes were not yet under their optimum load. A practical aspect, which is not shown by the results, is the machinability of SAC. The SAC cathodes were relatively fragile because the material was cut from an existing water purification cartridge. The fragility of thin SAC cathodes was essentially due to the relatively large activated‑carbon particles (≈0.5–1 mm). This fragility prevented to strongly tighten the cathodes onto the current collector. This could explain why more power was produced during the temporal phase (≈1050 μW), under 300 Ω, than during the polarisation experiment (≈930 μW; Fig. 2a). Hence, under their tested form, the SAC cathodes had a good performance to cost ratio but a low robustness. For this reason, the SAC cathodes were not tested further. Contrarily, the SSM-MFCs mounted with PVA-10 cathodes were the less performing under continuous loading (Fig. 2a). However, results from the polarisation curves showed that SSM-MFC incorporating PVA cathodes could produce higher power levels (1320 μW @ 100 Ω; Fig. 2b), nearly on par with the PTFE cathodes (1478 μW @ 100 Ω; Fig. 2b).

**Fig. 3:**
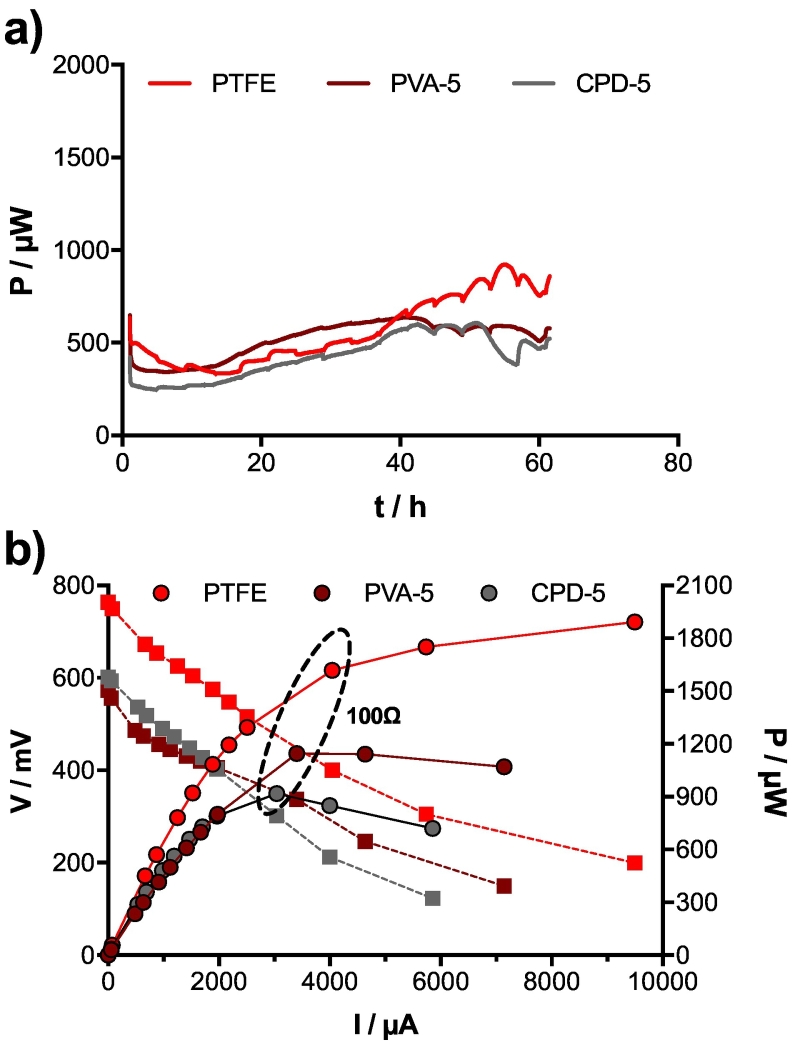
Electrical performance of the SSM-MFCs of the second experimental batch. a) Temporal evolution of the power produced under 300 Ω load. *T* = 0 starts once the load was applied, 3 days after inoculation under OCV conditions. b) Results from the polarisation experiment carried out at the end of the inoculation phase (Day 6).

Wei et al. have shown that binder loading does not affect significantly the cathodes performance [45]. It was therefore decided to decrease the binder concentration of the tested PVA and CPD cathodes during the second experimental batch. The hypothesis being that a higher concentration of activated carbon in the mix would increase its electrical conductivity and performance. Moreover, also a decrease in the binder content might lead to lower the overall cost and be therefore beneficial. At the end of the inoculation phase of the second experimental batch, SSM-MFCs mounted with PTFE cathodes were again the highest performing amongst the investigated cathodes (≈875 μW; Fig. 3a). At that stage, the performance of the SSM-MFC mounted with either PVA-5 or CPD-5 cathodes were on par (≈580 μW; Fig. 3a). The polarisation experiment showed that the SSM-MFCs containing PVA-5 cathodes had the potential to produce roughly 25% more power than the MFC using CPD-5 cathodes (Fig. 3b). If the protocol of the CPD-5 fabrication allows for much faster production rates, an organic solvent was needed to dilute the binder (PlastiDip) hence allowing for its homogeneous distribution. However, as organic solvents are adsorbed by activated carbon [46], the surface area of the CPD-5 cathodes could have been affected, thus being the cause for the observed lower power production (Fig. 3b).

**Fig. 4:**
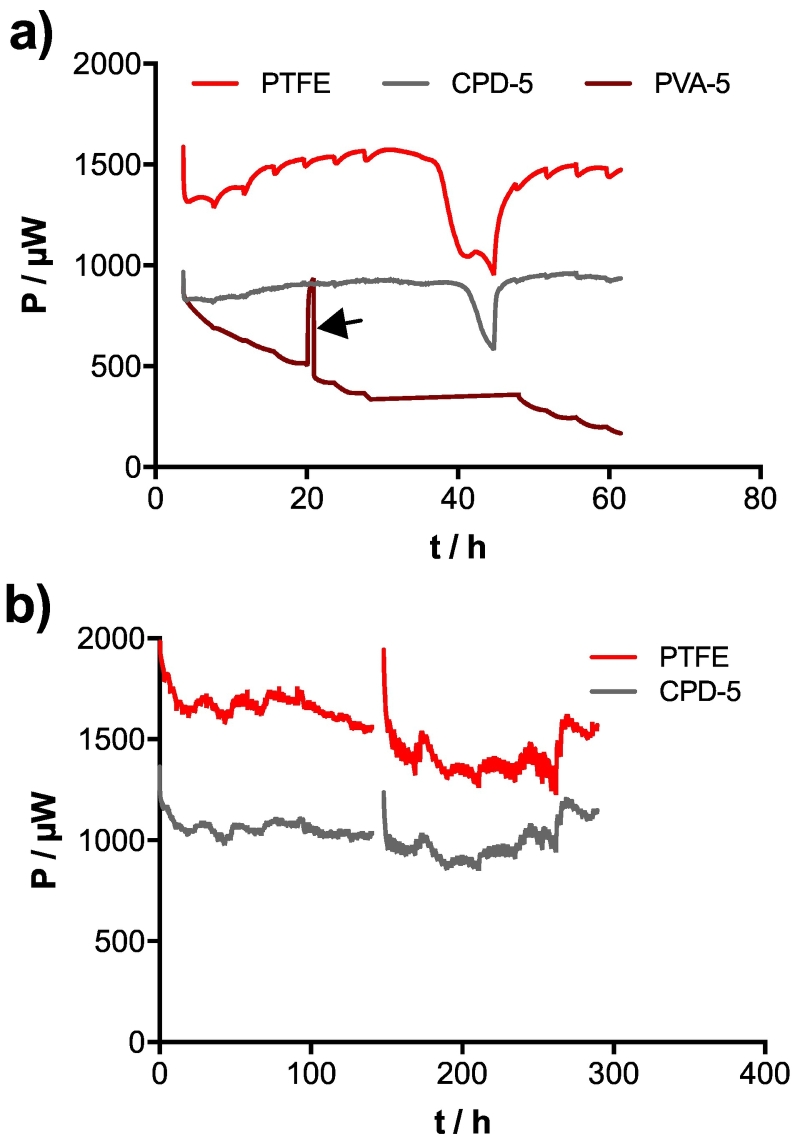
Electrical performance of the SSM-MFCs after the inoculation phase. a) Temporal evolution of the power produced under 200 Ω load. b) Temporal evolution of the power under 100 Ω. Arrow indicates when the cathode connections were tightened.

**Fig. 5:**
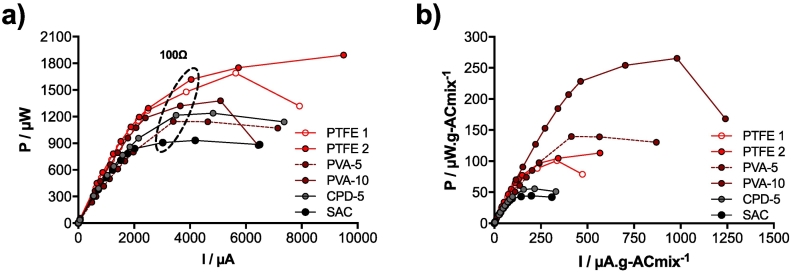
a) Power curves of all the tested cathodes after running 13 days. Due to its instability, the plotted PVA-5 results are from 3 days (Fig. 3b). The two PTFE curves illustrate the repeatability of the results despite being different runs and materials. PTFE 1 stands for the first run (Fig. 2b) and PTFE 2 stand for the second run (Fig. 3b). b) Normalisation of the data by the carbon loading.

PVA-5 cathodes were able to produce more power than the CPD-5, but these performance levels were not stable over time. As illustrated by the results, the power of the SSM-MFC using PVA-5 cathodes drastically decreased (Fig. 4a). This decrease might be due to a physical degradation resulting in a loose connection between the cathodes and the current collector. Retightening the connection resulted in a power increase on par with the SSM-MFCs mounted with CPD-5 cathodes. However, the connection was not stable since the SSM-MFC having these cathodes continued degrading. This degradation could be down to two possible factors: either the binder concentration (PVA) was too low, or the fixation of the PVA at 180 °C was not done properly (e.g. resulting in dissolution of PVA). Compared to the PVA-5 cathodes, the PTFE and the CPD-5 cathodes were stable over the whole duration of the experiment (Fig. 4b). The temporal run confirmed that 100 Ω was the optimum load for these setups: the power outputs were comparable to the power indicated by the results of the polarisation experiments (Fig. 5a).

Comparing all the power curves indicates that the PTFE cathodes were the best performing cathodes amongst the tested ones (Fig. 5a). SSM-MFCs mounted with PVA and PlastiDip (CPD) based cathodes were producing similar level of power (Fig. 5a). Amongst the PVA cathodes (e.g. PVA-10 and PVA-5), the binder concentration did not seemed to drastically influence power outputs (Fig. 5a), which further support the results form Wei et al. [45].

The power output was also normalised by the mixture loading that lead to new considerations related to the performance of the tested cathodes binder. From the cost perspective, PVA is more efficient than the PTFE since more power is produced per amount of loaded mixture (Fig. 5b; Table 2). Of particular importance is the fact that PVA (≈2 $ kg^−1^) is much cheaper than 60% PTFE dispersion (≈6 $ kg^−1^). Even though CPD cathodes were on par with the PVA ones in terms of absolute power, from a cost perspective, CPD were less efficient as cathode material (Fig. 5b; ≈5–8 $ kg^−1^). Regarding the power produced per amount of material used, the SAC cathodes were the least efficient (Fig. 5b), but also the least expensive material (0.3–3 $ kg^−1^). A previous study investigating PTFE alternatives has shown, with a different MFC design (i.e. ceramic membrane), that chitosan was a good alternative [47]. The MFCs mounted with chitosan based cathodes were producing 40% less power (i.e. maximum power) than the PTFE control whilst using only 2.5% of binder [47]. Compared to these results, the PVA-10 cathodes seem to be a good alternative since they were producing 85% of the maximum power produced by the control (Fig. 5a).

**Table 2:** Cost evaluation of each cathode material for the production of 1 W of energy.

Cathodes Material	Power density^a^	Area needed for 1 W	Loading	Quantity AC for 1 W	Quantity binder for 1 W	Cost AC	Cost binder	Cost current collector	Energy cost
Units	mW·m^−2^	m^2^.w^−1^	mg·cm^−2^	kg	kg	$	$	$	$·W^−1^
PTFE	101	9.9	186	6.91	3.84	14.10	23.39	65.35	102.84
SAC	51	19.6	236	23.14		69.42	–	–	69.42
PVA-10	73	13.7	56	3.45	0.38	7.05	0.87	90.41	98.33
PVA-5	64	15.6	91	6.75	0.36	13.79	0.81	103.13	117.73
CPD-5	68	14.7	248	17.32	0.91	35.37	6.38	97.06	138.82

a Normalized by the total exposed surface area of both cathodes of each SSM-MFC (2 cathodes = 45 cm^2^ × 4 sides = 180 cm^2^). It has to be noted that the quantity of material was calculated for the surface area of the 2 cathodes (90cm^2^).

Although PVA binder would be a good alternative to PTFE, the instability of PVA cathodes over time, as observed with the PVA-5 cathodes (Fig. 4a), indicates that further improvements need to be made to improve its structural integrity. Nonetheless, due to its lower cost and biocompatibility PVA binder is an alternative that would need to be pursue. When balancing production cost (i.e. labour and material) to the produced power, results clearly indicate that CPD cathodes would not be an interesting option (Table 2). The cost to power ratio of the SAC is conversely interesting. Indeed, SAC cathodes do not need stainless steel backbone and could be produce to shape at low cost (Table 2). It has to be noted that microbial fuel cell technology is not only a low-level power source, it is also a wastewater treatment process.

## 4 Conclusions

The sintered activated‑carbon cathodes present high performance to cost ratio as they are self-supported and could be easily mass manufactured. However, the tested material would need a composition change to increase its robustness. Such improvement should focus on utilizing smaller particle size to have a more homogenous repartition of the HMW-PE binder at the scale of the used cathodes (3 mm thick). Between CPD and PVA binder, results clearly demonstrated that PVA based cathodes were the only one that would be a good alternative to PTFE-based cathodes. The low cost and biocompatibility of PVA further support that conclusion. Unfortunately, their stability over time was low and their production protocol would need to be further improved. For the whole duration of the experiments, and amongst all the tested cathodes, SSM-MFCs mounted with PTFE cathodes were the more powerful. Hence, results demonstrated that at present and with regards to the specific SSM-MFC design and power production, PTFE are still the most suited binder for activated carbon based cathodes.
